# Regulatory T Cells in Allergy and Asthma

**DOI:** 10.3389/fped.2017.00117

**Published:** 2017-05-23

**Authors:** Elena Martín-Orozco, María Norte-Muñoz, Javier Martínez-García

**Affiliations:** ^1^Department of Biochemistry and Molecular Biology B and Immunology, School of Medicine, Murcia Biohealth Research Institute-University of Murcia (IMIB-UMU), Regional Campus of International Excellence “Campus Mare Nostrum”, Murcia, Spain

**Keywords:** regulatory T cells, allergy, FOXP3, asthma, immune system, inflammation, tolerance, children

## Abstract

The immune system’s correct functioning requires a sophisticated balance between responses to continuous microbial challenges and tolerance to harmless antigens, such as self-antigens, food antigens, commensal microbes, allergens, etc. When this equilibrium is altered, it can lead to inflammatory pathologies, tumor growth, autoimmune disorders, and allergy/asthma. The objective of this review is to show the existing data on the importance of regulatory T cells (Tregs) on this balance and to underline how intrauterine and postnatal environmental exposures influence the maturation of the immune system in humans. Genetic and environmental factors during embryo development and/or early life will result in a proper or, conversely, inadequate immune maturation with either beneficial or deleterious effects on health. We have focused herein on Tregs as a reflection of the maturity of the immune system. We explain the types, origins, and the mechanisms of action of these cells, discussing their role in allergy and asthma predisposition. Understanding the importance of Tregs in counteracting dysregulated immunity would provide approaches to diminish asthma and other related diseases in infants.

## Regulatory T Cells (Tregs): Types and Phenotypic Characteristics

Regulatory T cells are a specific CD4^+^ T cell population involved in peripheral tolerance by inhibiting autoreactive CD4^+^ T cells that have eluded negative selection in the thymus and controlling inflammation in diverse biological processes, such as infection, metabolic disease, tissue repair, cancer, and hypersensitivity reactions (1, 227). Tregs suppress inflammation by upregulating immunosuppressive molecules and tissue homing receptors and repressing genes, preventing the acquisition of pro-inflammatory functions (2). At least five subtypes of Tregs have been identified and classified based on the expression of the transcription factor FOXP3. The group of FOXP3^+^ Tregs includes thymus-derived Tregs (tTregs) and peripheral regulatory T cells (pTregs). The FOXP3^−^ Tregs group includes Tr1, Th3, and CD8^+^ Tregs.

### FOXP3^+^ Tregs: tTregs and pTregs and Their Phenotypic Markers

Two types of CD4^+^FOXP3^+^ Tregs have been described. A major population of Tregs of thymic origin, called thymus-derived Tregs (tTregs), also known as natural Tregs (nTregs), which mediate tolerance to self-antigens (3), and a second population that arises extrathymically in secondary lymphoid tissues when naive T cells (Tconv) encounter antigens and differentiate under the influence of TGF-β (4). These peripheral regulatory T cells (pTregs) are mainly present in the gastrointestinal tract and in the lungs during chronic inflammation, with specificities directed against microbial antigens or environmental allergens (5). Probably due to their different origins, FOXP3^+^ tTreg and pTreg cells are characterized by a non-overlapping T-cell receptor (TCR) repertoire. Based on that, a division of roles has been suggested in which tTreg would regulate immune responses developed against self-antigens and pTreg cells would regulate immune responses against “non-self” infectious or innocuous antigens (6, 7).

At the moment, there are no exclusive markers for Tregs, although it has been described that Tregs express several molecules that altogether characterize them and allow their identification in comparison to T conventional or effector T cells (Teff).

Thus, CD25 was the first marker associated with Tregs; nevertheless, this protein is also present in recently activated T cells. As a consequence, CD25 expression can be used only to differentiate Tregs from Tconv. In naive mice, Tregs show constitutive expression of CD25, while in humans, Tregs exhibit very high levels of CD25, and activated T cells show intermediate expression of this molecule (8, 9).

FOXP3, a member of the forkhead transcription factor family, was identified as a Tregs-specific transcription factor in mice (10, 11) and in humans (12). More than 90% of murine Tregs express this transcription factor, while naive and Teff do not present detectable levels of this molecule. Similarly, most human CD4^+^CD25^high^ Tregs express FOXP3, but, contrary to the results observed in mice, human Teff express intermediate levels of FOXP3 upon activation for a short period of time (13), introducing serious doubts regarding the specificity of FOXP3 as an exclusive marker for human Tregs (14). Moreover, FOXP3 plays a decisive role in Treg cell lineage establishment and function (10, 11). With regard to that, a mutation in the *FOXP3* human gene is responsible for the human syndrome known as immunodysregulation, polyendocrinopathy, and enteropathy X-linked syndrome (IPEX), or X-linked autoimmunity and allergic dysregulation syndrome (XLAAD), equivalent to the murine syndrome known as Scurfy (10, 15–17). Murine and human diseases are characterized by low levels of circulating Tregs, suggesting a critical role for *Foxp3* and *FOXP3* for appropriate Treg differentiation in both species, respectively. Although 60–70% of patients with IPEX have mutations in FOXP3 and produced normal levels of IL-10 (18), other studies (19, 20) have described that certain IPEX patients lacked expression of CD25 (IL-2 receptor alpha chain) and showed defective IL-10 production after *in vitro* stimulation of their Tregs (20). These data suggest fundamental and non-overlapping roles for both Tregs (FOXP3^+^ and IL-10^+^) in the control of autoimmune and allergic disorders (9, 21).

*FOXP3* gene expression is regulated by epigenetic modifications of conserved non-coding sequences (CNS) presented in four elements. Regarding that, it is known that pTreg cells are less stable than tTreg cells and can lose FOXP3 expression and produce cytokines, such as IFN-γ and IL-17, under inflammatory conditions (22). This lack of stability can be explained by the methylation status of the CNS2 region of the *FOXP3* gene, which is stably hypomethylated in tTreg cells, but is incompletely demethylated in pTreg cells (23, 24).

In addition to CD25 and FOXP3, tTreg and pTreg cells express similar levels of shared Treg cell markers, such as cytotoxic T-lymphocyte antigen 4 (CTLA-4), glucocorticoid-induced TNFR-related protein (GITR), inducible T cell Costimulator (ICOS), and CD103. However, many of those markers are also upregulated by activated CD4^+^ T cells under inflammatory conditions, and their expression does not allow discrimination between these two populations (25). In order to distinguish between tTreg and pTreg cells, the use of Helios and Neuropilin-1 (Nrp-1) has been proposed since the expression of such markers is higher in tTreg compared with pTreg cells (26–28). Finally, thymic-derived Tregs can be differentiated into two subpopulations based on the degree of FOXP3 expression and the presence or absence of CD45RA (29). These populations are “*CD25*^++^*CD45RA*^+^*(FOXP3^lo^) resting Treg cells (rTreg cells) and CD25*^+++^*CD45RA*^-^*(FOXP3^hi^) activated Treg cells (aTreg cells), which represent different stages of Treg cells differentiation and are both suppressive in vitro*” (29).

As already mentioned, it seems that Tregs display a certain level of functional plasticity since they have the capability to perceive cytokines in their milieu and respond to them with the expression of a subset of appropriate genes; this functional plasticity is critical for the regulation of the peripheral immune response. In fact, Tregs are widespread in non-lymphoid tissues, skin, lungs, and intestine, since barrier tissues contain large populations of specialized Tregs that utilize several of the same molecules to reach the same sites as their effector cell counterparts. Thus, specific transcription factors activation and the expression of several chemokine receptors such as CCR4, CCR6, CXCR3, and CXCR10 have been suggested to characterize four different subsets of human tTregs (30), which colocalize and control specific Th subsets (Th1, Th2, Th17, Th22) expressing identical chemokine receptors. Supporting this idea, it has been shown that Treg cell homing to the skin requires the expression of CCR4 by Tregs and of ligands for P- and E-selectin by cutaneous vascular endothelial cells. In fact, Tregs with deficiency in one of these molecules expression fail to correctly control immune responses in the skin (31, 32).

#### Generation of Thymus-Derived Regulatory T Cells (tTreg)

It is well established that thymocyte destiny depends on the binding affinity/avidity of the TCR to its self-peptide–MHC ligands on antigen-presenting cells (APC). Thus, although thymocytes expressing a TCR with low affinity for self-peptide–MHC complexes are positively selected and differentiate into conventional T cells, thymocytes that bind self-peptide–MHC complexes with high affinity undergo negative selection and experience cell death in order to remove potentially self-reactive T cells. A third possibility is the existence of thymocytes expressing TCRs with intermediate avidity for self-peptide–MHC, which may differentiate into cells with a regulatory function (tTregs) (33).

Together with the interaction of the TCR with self-peptide–MHC, additional signals are required for thymic induction of Tregs, such as costimulation through CD28 or the signaling provided by the IL-2R-γ_c_ cytokine family. Thus, deficiency of tTregs was observed in CD80/CD86 and CD28 knockout animals (34). Accordingly, the absence of the Treg population from the thymus and periphery was observed in IL-2R-γ_c_ knockout animals (35) as well as the emergence of autoimmunity in animals lacking IL-2Rβ, which could be repaired by the transfer of Tregs from control mice (36, 37). Furthermore, in IL-2^−/−^ or CD25^−/−^ mice, the expression of FOXP3 in thymocytes was drastically reduced and these animals developed fatal autoimmunity disorders (35).

Regulatory T cells may also be induced to differentiate at a double-positive (CD4^+^CD8^+^) developmental stage (38), since a momentary reduction of FOXP3^+^ thymocytes in newborn mice was observed upon elimination of TGF-βRI in the murine thymocytes at a double-positive (CD4^+^CD8^+^; DP) development stage (39). These results support the hypothesis that thymic Treg selection is enhanced by TGF-β signaling. In this regard, TGF-β signaling has been demonstrated to control apoptosis of self-reactive thymocytes through a Bim-dependent mechanism, raising the Treg precursor cells (40). In addition to the above data in mice, studies on the development of human thymic Tregs have shown that an important percentage of FOXP3^+^ single-positive (SP) cells derive from FOXP3^+^ DP thymocytes (41).

In summary, the majority of the Tregs originate in the thymus at the CD4 SP stage and intermediate signal strength TCR, IL-2, CD28, and TGF-β are needed for FOXP3 upregulation during the process of Treg cell generation. Additionally, Tregs may be differentiated at a previous double-positive stage in the presence of TGF-β.

#### Generation of Peripheral Regulatory T Cells (pTreg)

Experiments based on the transfer of purified FOXP3^−^ CD4^+^ T cells into lymphopenic mice followed by the subsequent analysis of the TCR repertoire of the FOXP3^+^ T cells generated, compared with that of cells which remained FOXP3^−^ demonstrated that “*TCR repertoires were only partially overlapping by FOXP3^+^Tregs and FOXP3^−^ non-Treg CD4^+^T cells present in control mice*” (42, 43). Furthermore, it was found that the TCRs from Tregs located in the colon were different from those expressed by Tregs from other tissues, and a subset of these colonic TCRs was specific for antigens associated with the gut microbiota (44). The above results suggest that while the differentiation of thymic Tregs is induced by intermediate affinity interactions of TCRs with self-peptide–MHC molecules, the development of peripheral Tregs is probably promoted by encounters with foreign antigens, e.g., foods, gut microbiota, and allergens.

Nevertheless, it has been suggested that the TCRs expressed by pTreg cells probably exhibit high affinity, as shown by the observation that “*rare high-affinity antigenic peptides allow for most efficient FOXP3 induction upon stimulation of a cognate transgenic TCR displayed by peripheral CD4^+^T cells in comparison with a less efficient pTreg generation by low-affinity peptide variants*” (45). Additionally, it seems that insufficient activation of T cells promotes the induction of FOXP3, since CTLA-4 expression is essential for the induction of FOXP3 *in vitro* by a mechanism dependent on TGF-β presence (46), while CD28 has the contrary effect (47, 48). Thus, *in vitro* and *in vivo* studies suggest that FOXP3 induction and pTreg cell generation require high-affinity TCR signaling together with suboptimal costimulation (high CTLA-4 and low CD28 signaling) (40), and the process is helped by the presence of high amounts of TGF-β (47). Signaling through TGF-βR seems decisive for the expression of FOXP3 in most peripheral CD4^+^ T cells (49).

The pTreg cell generation requires the combined action of soluble factors, such as TGF-β and IL-2, in the microenvironment and the presentation of the antigens by appropriate APCs. Furthermore, the presence of all-transretinoic acid (ATRA) in the Tconv environment synergizes with TGF-β, and this effect is great enough to promote pTreg generation even when a high costimulation is being produced. This is particularly evident in lung tissues where resident macrophages (CD45^+^CD11c^+^MHCclass II^low^F4/80^+^) constitutively expressing TGF-β and retinoic acid are the main subset of cells driving pTreg cell induction from naive CD4^+^ Tconv cells (50).

The data discussed so far indicate that pTreg cells generation is influenced by a specific type of TCR signaling, and costimulation, and through cooperation with other signals, such as TGF-β, IL-2, and ATRA. These conditions suggest that pTreg cell differentiation could be restricted to precise locations such us mucosal surfaces where they may regulate immune responses to harmless antigens such as commensal microbiota and prevent allergic inflammation. Supporting these ideas, “*studies using Rag-1-deficient T-B monoclonal mice sufficient or deficient in FOXP3 demonstrated that TCR transgenic pTreg cells were sufficient to establish mucosal tolerance and control allergic inflammation induced by the model antigen recognized by the TCR*” (5).

Additionally, several studies suggest a role of microRNAs (miRNAs) in the generation and function of Tregs (51, 52). Specifically, miR155 was recently described as a player in Treg cell differentiation and was shown to induce upregulation of several Treg cell-associated genes when transferred to conventional T cells (51, 53). It has also been reported that an acquired decrease of the endoribonuclease Dicer, involved in the generation of miRNAs (54), induces spontaneous autoimmunity in a disease model (52). The progression to autoimmune disease was associated with a marked decrease in Dicer and an increased miR-155 expression which can upregulate CD62L in Tregs. Furthermore, Singh et al. (55) found three miRNAs (miR-15b/16, miR-24, and miR-29a) that regulated the induction of Tregs from naive CD4^+^ T cells, with miR-15b/16 having the highest effect. Important genes regulated by miR-15b/16 were Rictor and mTOR, which encode components of the mTOR signaling pathway involved in controlling the generation of Tregs.

In summary, the requirements to promote induction of pTregs include high-affinity interaction of TCRs with peptide: MHC complexes; suboptimal activation of dendritic cells (DCs); mucosal administration of peptide; signaling by cytokines, such as TGF-β and IL-2; and regulation by the appropriate miRNAs (49, 51–53, 55, 56).

### FOXP3^−^ Tregs: Tr1, Th3, and CD8^+^ Tregs

In addition to FOXP3^+^ Tregs residing in lymphoid and non-lymphoid tissues, there are other tissue-residing Tregs with unclear origins. They could have a thymic origin and migrate to peripheral tissue and proliferate in response to inflammation or they could develop from CD4^+^CD25^−^ Tconv as a consequence of antigen recognition in the tissue.

Type 1 regulatory T cells (Tr1) are a population activated in the periphery after antigenic stimulation in the presence of IL-10 (57, 58). These cells are characterized by the expression of CD4, CD226, lymphocyte-activation gene 3 (LAG3), and CD49b as their specific markers (59). The Tr1 Tregs produce a large amount of the “*cytokines IL-10 and TGF-*β, *some IL-5, low levels of IFN-*γ, *and IL-2, but no IL-4*” (57). The secretion of the immunosuppressive cytokine IL-10 is the main mechanism by which Tr1 cells are thought to regulate the immune response (60).

T helper type 3 cells (Th3) is a subset of Tregs that differentiate in the periphery upon interaction with a specific antigen and mediate suppression by secreting the cytokine TGF-β. These cells “suppress the proliferation and activation of Th1 cells and the development of autoimmunity in the mouse model of multiple sclerosis” (61). Thus, Th3 cells may have a role in controlling autoimmunity and allergy in humans (62), although their role in the maintenance of immune tolerance in humans has yet to be clearly defined (60).

An immunoregulatory function has also been attributed to some CD8^+^ Treg subsets, and blocking this immunosuppressive action may induce autoimmune reactions. The most frequent phenotype for CD8^+^ Tregs is CD25^+^CD28^−^ (63, 64), although the expression of other markers has been described and includes CTLA-4, CD122, CD38-4, GITR, CD8αα, and CD103. This Treg subset has been observed in tonsils but is infrequent in peripheral blood. The action mechanisms of these cells include suppression by cell contact; the release of regulatory cytokines such as IL-10 and TGF-β; and the promotion of anergy in APCs (60, 65).

## Immune Maturation and Allergy/Asthma Incidence

In addition to genetic and environmental factors, proper immune system maturity during the first years of life is fundamental to avoid allergic asthma (AA). In fact, a high percentage of asthmatics (ninety percent) are diagnosed by 6 years of age, suggesting that the influence of intrauterine milieu and early life events such as atopic diseases and wheezing illnesses induced by respiratory viral infections are highly determinant for the development or not of asthma during childhood (66, 67). In this sense, abnormal regulatory T-cell function and/or numbers have been pointed to as the main cause of AA incidence and it has been observed that Tregs are already defective in the umbilical cord blood of newborns at a genetic risk of allergy (67, 68).

### Asthma: Definition and Classification

Asthma is the most frequent childhood chronic inflammatory airway disease worldwide and is characterized by reversible airflow obstruction. The prevalence of asthma in children ranges between 5 and 20%, with approximately 300 million people suffering from this disease (69, 70).

Asthma can be classified as mild, moderate, or severe according to National Asthma Education and Prevention Program, Global Initiative for Asthma (GINA), or American Thoracic Society (ATS) guidelines (71, 72). Nevertheless, asthma is an extremely heterogeneous disease that develops in many clinical forms or phenotypes with distinct pathogenic mechanisms and is induced by diverse sensitizers such as allergen exposure, viral infection, oxidative stress, and air pollution, amongst others. Today, clinical asthma is mainly divided into two main phenotypes: AA and non-allergic asthma (NA). AA is characterized by airway hyperreactivity (AHR), eosinophilic airway inflammation, increased total and specific IgE levels and blood eosinophil counts, elevated mucus production, and reversible airway obstruction and remodeling (73). Th2 cells initiate AA, and the characteristic cytokines are produced by both Th2 cells (IL-4, IL-5, and IL-13) and type 2 innate lymphoid cells (ILCs) (IL-5 and IL-13). These molecules induce IgE class switching by B-cells (IL-4), eosinophils infiltration (IL-5), and hyperplasia of goblet cells (IL-13) (74).

**Figure 1:**
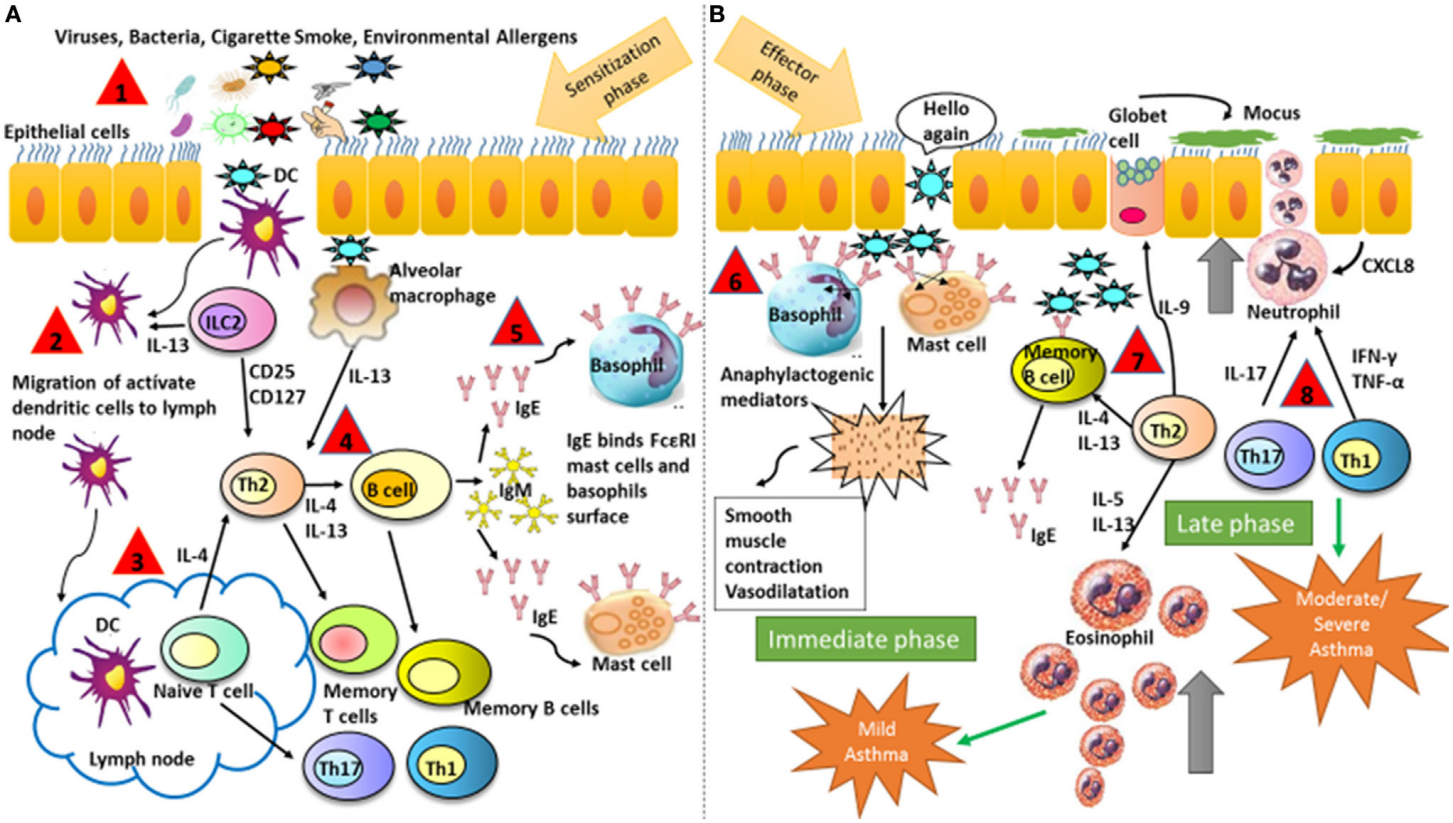
**Schematic illustration representing major mechanisms that contribute to the development of asthmatic symptoms**. **(A)** Sensitization and memory phase. The sensitization phase is initiated when the allergen is introduced in the organism for the first time. At the end of this phase, the immune system is prepared for a new contact with the same allergen. **(B)** Effector phase includes the immediate and late responses. The effector phase is initiated after a new contact with the same allergen that binds to IgE on the surface of basophils and mast cells. The followed numbers describe the consecutives steps in asthma: (1) antigen entry, (2) migration of dendritic cells from the alveolar space to the lymphatic node, (3) antigen presentation to naive T cell and maturation into Th2, (4) production of Th2 cytokines and antibodies production, (5) isotype switch from IgM to IgE, (6) IgE binds to FcεRI on mast cells and basophils and inflammatory mediators are released, and (7) and (8) response mediated by memory B-cells and Th cytokines with recruitment and activation of eosinophils and neutrophils. DCs, dendritic cells.

Allergic symptoms usually start in childhood upon sensitization of the airways to common allergens such as dust mites, animal dander, or tree pollens (Figure 1, Step 1). The immune mechanisms involved are divided into two phases. First, a sensitization and memory phase develops (Figure 1A). Second, the effector phase occurs and includes the immediate and late responses (Figure 1B) (75). Throughout the sensitization phase, the clonal expansion and development of allergen-specific Th2 cells and the production of the cytokines IL-4 and IL-13 take place (Figure 1, Steps 2–4). These cytokines induce B-cells class-switching to allergen-specific IgE antibodies, which bind to the high-affinity receptor for IgE (FcεRI) that is expressed on mast cells and basophils (Figure 1, Steps 4–5). The initiation of the effector phase takes place after a new encounter with the same allergen that binds to the IgE–FcεRI complex causing cross-linking and as a consequence triggering the activation of mast cells and basophils, which release the anaphylactogenic molecules contained in their cytosolic granules (Figure 1B, Step 6). These molecules trigger the symptoms of the immediate reaction (Figure 1B). Late-phase reactions are generated by the prolonged presence of the allergen, which initiates a specific Th2 cells activation, with the production of cytokines such as IL-4, IL-5, IL-9, IL-13, and IL-31. These are critical to sustaining specific IgE levels, the arrival of inflammatory cells to tissues, eosinophilia, mucus release, and smooth muscles contraction (76, 77). Such events can conclude with the more severe symptoms of allergy, such as asthma, rhinitis, dermatitis, and less frequently systemic anaphylaxis. Additionally, recently identified cytokines such as IL-25, thymic stromal lymphopoietin (TSLP), and IL-33, secreted by injured epithelial cells among others, have been associated with the development of the Th2 response (78, 79). Furthermore, additional T-cell subsets contribute to the allergic airway disease, such as IL-9-producing Th9 cells but also Th1 and Th17 cells (80). Finally, it has been found that neutrophils infiltrate airways after recruitment by certain allergens and release inflammatory mediators contributing to asthma development (81).

### Influence of Intrauterine Milieu in the Neonatal Immune System and Risk of Asthma Development

The intrauterine milieu is critical for an appropriate immunological maturation of the fetus but could be influenced by environmental factors. Thus, it has been shown that maternal conditions such as diet, microbiota, inflammation, and environmental antigens can be decisive for an adequate immune development during pregnancy and during the postnatal period. Therefore, allergy/asthma can represent an early consequence of inappropriate immune regulation (82).

The development of the fetal immune system and organs such as lungs and airways take place during the intrauterine period and they are more vulnerable to environmental influences. Microbial exposures during this period are particularly important. Thus, based on the hygiene hypothesis, it may be possible that microbial antigen transfer from the mother to the progeny starts during the pregnancy (83–85) dispensing a first supply of immune stimulation. Epidemiological research has also shown that “a high microbial environment during pregnancy induces greater protection from allergy than postnatal exposure alone” (82, 86). Thus, maternal exposure to microbial compounds during pregnancy is coincident with increased expression of certain Toll-like receptors (TLR2 and TLR4) and the CD14 molecule on peripheral blood cells, suggesting that this type of exposure during the prenatal period might prevent childhood sensitization (86). For example, maternal exposure to farm animals during fetal development is related with an improvement in the function and number of Tregs within umbilical cord blood and a reduction in the secretion of Th2 cytokines or lymphocyte proliferation upon innate stimulation (39). Additionally, experiments conducted in mice have demonstrated that exposure to endotoxin during pregnancy avoids future sensitization and lung inflammation episodes induced by allergens in the offspring (87). Moreover, a mother tolerant before pregnancy can transfer this immunological tolerance to her descendants, suggesting that the immunological status of the mother has a great influence in the immune response of the offspring to allergens (88). In fact, it has been shown that during fetal development, the tTreg levels in venous blood change depending on pet exposure levels and the atopic condition (65). In addition to infection and exposure to allergens, it has been demonstrated that pollution and diet influence the development of disease during early infancy (89). Thus, enhanced asthma symptoms in children have been associated with poor maternal intake of vitamins D, E, and zinc during fetal development (90). Specifically, vitamin D has been associated with FOXP3^+^ and IL-10^+^ Tregs generation and survival in humans and mice (91). Furthermore, it has been shown that diet-related factors can control the epigenetic mechanisms that modify the risk of allergic disease (89). Thus, a maternal diet high in folates, choline, methionine, vitamin B12, or exposure to cigarette smoke may promote DNA methylation, inhibit gene transcription, and foster asthmatic symptoms (92, 93). These studies have shown that the regulatory cytokines IL-10 and TGF-β participate in the dysregulated immunity of the lung.

### Postnatal Maturation of the Immune System and Risk of Asthma Development

High susceptibility to infections and/or the emergence of atopic and/or asthmatic manifestations in children have been linked to the functional immaturity of the immune system influenced by the degree of exposure to several immunostimulatory factors during embryonic development and during the early infancy (76). In general, the immune system that is still immature at birth and triggering most TLR ligands in their leukocyte subsets produces less type1 IFN, IL-12, and TNFα but increased amounts of IL-1, IL-6, IL-10, and IL-23 in comparison to the same adult cells; in general, the newborn innate response presents Th2- and Th17-biased immunity while Th1 response is scarce. This fact highlights the fundamental role of antigen exposure during early infancy for an adequate maturation of the immune system. Developmental deficiency in the circulating DC compartment has been implicated in atopic diseases in children (94), with attenuated capacity for IFN-γ and IL-12 production (95, 96) and with a disequilibrium in the balance between cytokines secreted by Th-cells (pro-inflammatory versus regulatory) (97). Furthermore, children at an elevated risk of atopy usually display decreased responses to certain vaccines (98). This could be explained by an immune dysregulation during the intrauterine period and/or an absence of exposure to infectious and/or environmental microbes during childhood. As a result, children may experience a failure in their protective Treg responses to allergens (99, 100). Additionally, it is possible that the lack of deviation from an allergen-specific Th2 immune response toward a Th1 may play a role (101). During childhood, such mechanisms may be alternatively activated by different types of microorganisms, albeit infectious or innocuous. For example, helminthic infections are related to IL-10 production and diminish the risk of allergic disorders (102) and have been shown to induce Tregs in an animal model of allergen challenge, thus preventing development of airway inflammation (103). A murine model has demonstrated that a heat-inactivated suspension of *Mycobacterium vaccae* also protected against airway inflammation *via* IL-10 and TGF-β production (104). However, the preventive effect of a livestock exposure may be through TLR-mediated immune bias toward Th1 responses to antigens present in the farm environment (105). In relation to that, it has been shown that the immunosuppressive role of CD4^+^ CD25^+^ Tregs may be regulated by TLR signaling during the course of the immune response. TLR signaling may influence the balance between CD4^+^ Th and Tregs and, as a consequence, orchestrate the subsequent immune response. In fact, a significant decrease in CD4^+^ CD25^+^ Tregs has been described in TLR2-deficient but not TLR4-deficient mice in comparison with control mice. Other data suggest that DC maturates *in vivo* upon binding of their TLR ligands, which subsequently regulate the development of the Teff (106).

In addition, the intestinal microbiota modulates the newborn Th2-biased immunity by promoting a Th1-cell response (107). Colonization of the newborn with the gut microbiota begins right after birth and is regulated by specific mucosal DCs, which bind antigens and favor T effector or Tregs differentiation. These DCs are characterized by a high expression of TLRs and costimulatory molecules upon interaction with TLR ligands, by production of the immunosuppressive cytokine IL-10 and absence of pro-inflammatory factors (108). It can be hypothesized that this specific control of TLR responsiveness is a mechanism to prevent unwanted inflammatory responses.

Interestingly, infection with a specific type of bacteria has also been associated with Treg development. Thus, infection of newborn mice with *Helicobacter pylori* protects from the progression of allergic airway symptoms and has been related with an accumulation of Tregs in the lung (109). Furthermore, DCs exposed to the bacteria were impaired in their maturation after lipopolysaccharide stimulation and induced the expression of FOXP3 in Tconv (110).

Finally, one study demonstrated that environmental antigens were transferred from the mother to the lactating mice *via* breast milk. As a consequence of this, protection from AA and tolerance mediated by Tregs and TGF-β production were observed in such newborn mice. These data propose a mechanism explaining breast feeding-induced tolerance in neonates and support the role of maternal factors on the regulatory responses that affect the predisposition to allergic disorders (67, 111).

## Role of Tregs in Allergy and Asthma

### Importance of Tregs in Allergic Diseases

Although the population is continuously being exposed to a wide range of allergens, not everyone develops allergic sensitization, and not all atopic individuals are asthmatic. This fact could be explained by genetic susceptibility to both atopy and asthma and differences in the maturation of the immune system and organs/tissues affected by allergic diseases. With regard to immunological sensitization, it has been shown that under certain conditions non-atopic healthy individuals develop Th2-cell responses to common allergens. In this sense, the control of potentially harmful T cells requires the use of an active immunosuppressive mechanism by Tregs. Defective or overwhelmed suppression by Tregs could explain the development of allergic airway inflammation in asthma (105). Accordingly, the development of allergic reactions can result from decreased induction, impaired function, or both, of allergen-specific Tregs in genetically allergy-prone subjects. On the contrary, in another study, Treg numbers were higher in asthmatic versus healthy children, and Tregs of children with AA show sufficient suppression of Th1/Th2 cytokines; whereas Tregs from infants with NA do not. These results suggest that the high number of Tregs in certain patients with AA might still not been sufficient to control the disease or additional mechanisms, such as deficiency in innate immune regulation, may be relevant for persistent inflammation (70, 73).

Nevertheless, studies on human newborn Tregs have found an association between decreased regulatory function at birth and the development of allergic diseases. For example, a defect was detected in the suppressive function of newborn Tregs in a child who was later diagnosed of egg allergy (68), and an inverse relation has been found between postnatal deficiencies in Treg numbers and/or function and development of allergy phenotypes during childhood (112). Regarding that, it has been shown that “*whereas the turnover and suppressor function of non-atopic infant’s Treg cells appears to increase with age, there is a delay in this process in atopic infants*” (113).

The importance of neonatal pTregs in asthma prevention is based on the observation that the cytokines IL-4 and IL-6 inhibit FOXP3 expression in naive CD4^+^ T cells. As a consequence, the generation of Tregs should be less efficient when it goes in parallel with conventional T-cells activation due to the presence of an allergen. However, if pTregs can be induced at early times, the tolerance mechanisms would promote expansion of the pTreg population (114).

One study showed deficient CD4^+^CD25^hi^ T cell numbers and function and decreased FOXP3 (mRNA) in the lungs of asthmatic children in comparison to healthy controls (115). Another study has shown that patients with asthma have normal numbers of CD4^+^CD25^hi^ and CD4^+^CD25^hi^ FOXP3^+^ Tregs in peripheral blood compared to healthy individuals, although the expression of the FOXP3 protein was attenuated (116). Conversely, inhibition of Th2-cell responses to allergens has been described by Tregs in healthy donors. Thus, depletion of CD4^+^CD25^+^ T cells from PBMC of healthy donors increased the proliferation and Th2 cytokines release in response to allergens as compared to whole PBMC cultures (117). In addition, passive transfer of allergen-specific Tregs can attenuate chronic airway inflammation induced by the allergen. Importantly, CD4^+^CD25^+^ Tregs also inhibited airway remodeling, and this might occur through an early decrease of the profibrotic cytokine TGF-β in lung (118).

### Activation and Recruitment of Tregs

An intense research focus concerns the study of the specific mechanism of Tregs generation and location in the airways of asthmatic mice and humans. The results of several studies suggest a role for ICOS-L-expressing DCs and the presence of the immunosuppressive cytokine IL-10 (119), although other researchers hypothesized that plasmocytoid DCs contribute decisively to the development of Tregs and accumulation in the airways (120). It has recently been reported that “*siglec-F^+^alveolar macrophages were found to be the major APC driving the differentiation of FOXP3^+^Treg cells in the lungs of mice following allergen inhalation, in a process requiring TGF-*β *and the retinal dehydrogenases, RALDH-1 and RALDH-2*” (121). The means by which Tregs migrate to the allergic lung tissue and lymph nodes of mice implicate the expression of certain chemokine receptors such as CCR4 and CCR7, respectively (122). Supporting this, studies have demonstrated that CCL17 and CCL22, both ligands for CCR4, have a role in the accumulation of CD4^+^CD25^+^ Tregs to the airway tissue throughout allergen response (118). The CD103^+^ conventional DC subset of the lungs (123, 124) is involved in the release of the CCR4 ligands, leading to the development and recruitment of Tregs in that location (125). Importantly, maintenance of T regulatory cell function required a continued allergen presence. In fact, discontinuous allergen exposure led to a reduction in Treg function and an increase of pathological symptoms (126). It would appear that Tregs control migration of effector cells to inflamed tissues and line up appropriate immune responses at different stages after antigen challenge.

### Mechanisms of Suppression by Tregs in Allergic Processes

**Figure 2:**
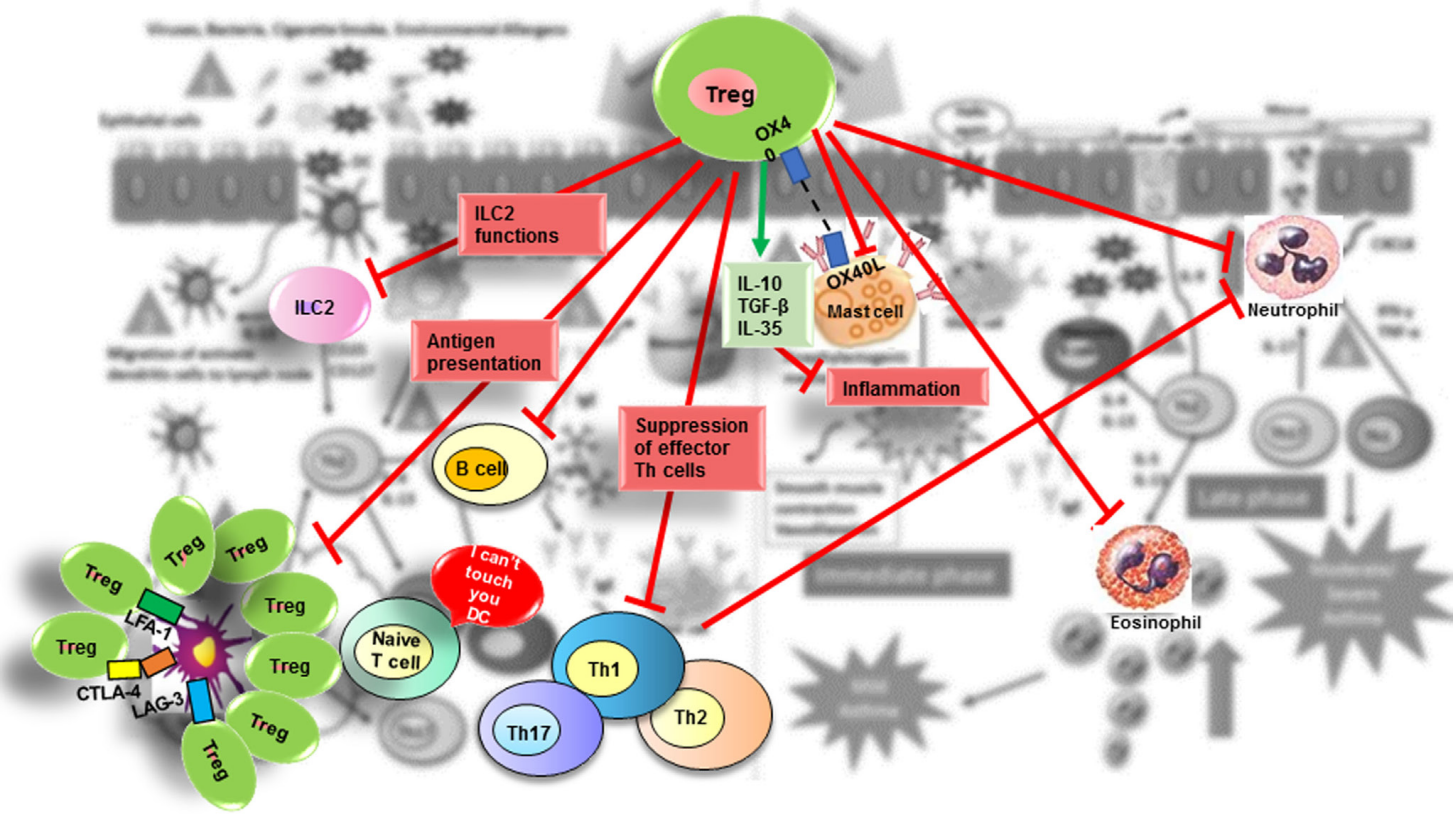
**Suppressive role of Treg in allergic diseases**. Tregs contribute to the control of allergic diseases through several pathways. Red arrows indicate suppressive effects that Treg exert directly or indirectly on effector cells (ILC2 cells; mast cells; basophils; DC; B-cells; effector Th2, Th1, and Th17 cells; neutrophils). The green arrow indicates suppressive molecules produced by Tregs.

Regulation is a general process that uses many strategies to attenuate inflammation, and the specific mechanism triggered depends on the tissue and associated milieu where the antigenic challenge occurs. Thus, Treg cell suppressive functions are similar when controlling autoimmunity or allergy and their action could be mediated by multiple mechanisms that involve either the release of suppressive cytokines (IL-10, TGF-β, and IL-35) (127–129) and cytolytic molecules [granzymes (Gzm) A and B] (130) or the downmodulation of APC through expression of inhibitory molecules such as CTLA-4 (CD152) and LAG-3 (CD223) (131); deprivation of trophic cytokines (IL-2 through CD25) (132); modulation of metabolic pathways (CD73 and CD39) (133); and modulation of the expression of specific transcription factors and receptors. Tregs function can also be regulated by endogenous danger signals or alarmins released by epithelial cells at the mucosal barrier. Colonic Tregs express the IL-33 receptor (ST2), allowing them to respond to the cytokine IL-33 produced by epithelial cells as a result of tissue damage. After IL-33 binding to their ST2 receptor, Tregs respond by amplifying their regulatory functions and restraining intestinal inflammation (28, 134). One of several combined mechanisms could be used by Tregs to regulate activation of the different cell types involved in the allergic response including B-cells, ILC2 cells, mast cells, eosinophils, neutrophils, CD4^+^ and CD8^+^ T cells, NK cells, NKT cells, monocytes, and DCs (135) (Figure 2).

#### Suppression of Type 2 ILC2 by Tregs

Innate lymphoid cells are a population of mucosal innate cells characterized by a lack of antigen specificity (absence of T- and B-cell receptors) and by shared developmental origin and phenotypic traits with T cells. ILC2 produce large amounts of Th2 cell cytokines and are linked to allergic disorders, such as asthma, chronic rhinosinusitis, and atopic dermatitis (28).

It has been demonstrated that peripherally induced Tregs effectively suppress the production of the ILC2-driven, pro-inflammatory cytokines IL-5 and IL-13, both *in vitro* and *in vivo* by blocking of ICOS:ICOS-L interaction on ILC2 cells. Inducible T cell Costimulator (ICOS) is a receptor expressed by ILC2 cells, Tregs, and others. It is well known that ICOS:ICOS-L interactions on ILC2 cells have a role in cell function and survival and are involved in controlling Th2 cytokine release, airway hyperreactivity (AHR), B-cell differentiation, and IgE class-switching in mice (136, 137). A recent study by Maazi et al. (138) demonstrated that the expression of ICOS and ICOS-L in ILC2s from human peripheral blood was increased by *in vitro* culture in the presence of IL-2 and IL-7 but not of IL-33. It has been demonstrated that cell contact is required for suppression of ILC2 mediated by Treg, TGF-β, and IL-10. Thus, *in vitro* stimulation of human ILC2s with IL-2, IL-7, and IL-33 and subsequent blockade of ICOS:ICOS-L interactions decreased the release of IL-5 and IL-13 cytokines (139). Additionally, it has been shown that human pTregs suppress syngeneic human ILC2s *via* ICOS-L to control airway inflammation in a humanized ILC2 mouse model (140) (Figure 2).

#### Suppression of Mast Cells by Tregs

Mast cells are essential to initiate the immediate phase of allergic reactions (Figure 1, Step 6), and their degranulation initiates the triggering of allergic symptoms (141). It has recently been demonstrated that the symptomatic phase of allergic disorders can be controlled by constitutive FOXP3^+^ Tregs in mice (142), although mast cells increased the IL-6 secretion. This inhibition by Tregs is produced *via* direct cell-to-cell contact between OX40 expressed on Tregs and OX40 ligand on mast cells, which leads to increased intracellular levels of cyclic AMP (cAMP) and results in blockage of extracellular Ca^2+^ (Figure 2). However, the suppression of IL-6 secretion by mast cells seems to be controlled *via* TGF-β (143). *In vitro* studies have shown that “*IL-4 and TGF-*β*1 had balancing effects on mast cell survival, migration and FcεRI expression, with each cytokine cancelling the effects of the other. Dysregulation of this balance may impact allergic disease and be an objective of targeted therapy*” (65, 144).

#### Suppression of APCs by Tregs

Dendritic cells are key initiators and master regulators of the allergen-specific immune response by processing and presenting antigens to Tconv and secreting cytokines that control T cell differentiation to a certain effector type. Tregs could directly act on DCs by downmodulating their surface expression of CD80/CD86 and subsequently blocking generation of an allergen-specific Th2 cell immune response. Suppression of DCs appears to be mediated through LAG-3, CTLA-4, leukocyte function-associated antigen 1 (LFA-1), and other molecules (Figure 2).

Thus, Tregs may express LAG-3, a homologous of the CD4 molecule that acts as a coreceptor of the MHCII complex, although with higher affinity. The binding of MHCII and LAG-3 reduces the maturation and costimulatory capacity of DCs and, as a consequence, diminishes their capacity for antigen presentation to Tconv (131, 145, 146). Activated human T cells express MHCII molecules, and interaction of Tregs *via* LAG-3 on Teff might also induce immunosuppression (147).

Additionally, murine and human Tregs exhibit a constitutive expression of the coinhibitory molecule CTLA-4, which is detected on the surface of Teff upon activation (148). The deficiency of the *CTLA-4* gene produces serious autoimmune disorders similar to those induced by defective *FOXP3*, demonstrating that CTLA-4 is essential for Treg function (149). CTLA-4 binds to the same ligands CD80/86 as CD28 (T cell costimulatory antigen) but with a higher binding affinity. The interaction between CD28 and its ligands CD80/86 on DCs is required for T-cell activation. However, CTLA-4 binding to the same ligands blocks such activation and induces the generation of anergic T cells. CTLA-4 may also suppress or decrease the surface expression of CD80/86 molecules, decreasing the activation of Tconv. Furthermore, the interaction of CTLA-4 with CD80/86 in Tregs can promote the generation of indoleamine 2,3-dioxygenase (IDO) that catalyzes degradation of the essential amino acid tryptophan to kynurenine, provoking the starvation of Teff and cell cycle arrest. Additionally, IDO induces pTreg generation (150). Also, CTLA-4 may be involved in the reduced glutathione synthesis observed in murine DCs, which promotes a Redox milieu unfavorable for the proliferation of conventional T cells (151). In relation to that, several studies have shown that certain polymorphisms in the *CTLA-4* gene are significantly associated with susceptibility to autoimmunity (152).

Regulatory T cells can control Tconv activation by reducing their interaction with DCs (153). In fact, it has been described that the aggregation of CD25^hi^ Tregs around DCs, *via* CTLA-4, downregulated CD80/86 molecules expression (149, 154). Thus, there is a competition between Tregs and Tconv for the interaction with DCs reducing their capability to activate Teff (105). This downregulation of CD80/86 expression by Tregs is also partly dependent on the adhesion molecule LFA-1, thereby indirectly impeding the activation of Tconv cells by APCs *in vitro* (155, 156) and *in vivo* (149, 157) (Figure 2).

Another surface molecule involved in immunosuppression is T cell immunoglobulin and ITIM domain (TIGIT), which is highly expressed on Treg and Teff. A protein named Poliovirus receptor (PVR, NECL5, or CD155), highly expressed on DCs and others, was identified as a high-affinity coreceptor for TIGIT. Upon interaction of Treg cell with DCs through TIGIT, secretion of the suppressive cytokines IL-10 and TGF-β by DCs was observed (158).

Neuropilin-1 is another molecule expressed by Tregs and which elongates their contact with DCs reducing the presentation of antigen to Tconv. These results were confirmed by using an anti-Nrp-1 antibody to abrogate Treg-mediated suppressive activity (159, 160). However, as other CD4^+^ cells express Nrp-1 (161) it is possible that the anti-Nrp-1 antibody was interfering with cell activation rather than Treg function (146).

Finally, ICOS:ICOS-L interactions between mostly plasmacytoid DCs and Tconv could result in their differentiation into IL-10-secreting Tregs (28).

#### Suppression of Th by Tregs

During the activation process, T cells follow several differentiation pathways, acquiring specific properties and functions. Th cells could differentiate into Th1 (IFN-γ-secreting cells), Th2 (IL-4/IL-5-secreting cells), Th17 (IL-17-secreting cells), and other subsets. Th1 cells are specialized in the elimination of intracellular microorganisms; Th2 cells are required for fighting extracellular pathogens; and Th17 cells protect against extracellular fungal and bacterial pathogens and have a role in autoimmune tissue injury. Regarding that, it has been shown that Tregs can avoid allergy by suppression of the effector Th1, Th2, and Th17 cells (162, 163) (Figure 2). Nevertheless, such results are not clear since it has been published that TGF-β1 secreted by FOXP3^+^ Tregs is necessary to block Th1 and support Th17-cell generation (164). On the other hand, it has been suggested that Tregs induced by nitric oxide can suppress Th17 but not Th1 cell development and function (165). Furthermore, it has been published that Tregs can also block the synthesis of the cytokine IFN-γ without inhibiting Th1 cell differentiation (166) and that FOXP3^+^ Tregs induce Th17 cell differentiation *in vivo* through IL-2 modulation (167). Additionally, it has been found that the cytokines IL-6 and TGF-β promote the generation of pathogenic Th17 cells (65, 168). Further studies should be performed to clarify the importance of Tregs in the suppression of specific Th subsets.

Recent evidence suggests the existence of a balance between Tregs and Th17 cells during the first stages of naive T cell development. Thus, the presence of IL-6 and TGF-β promotes the differentiation of Tconv into Th17 cells. However, without IL-6 in the milieu, T cells differentiate into Tregs. Additionally, IL-21 has a role in the generation of the Th17 subset and inhibits FOXP3. Given the contribution of Th17 cells in asthma, the inhibition of the Th17 population by Tregs is crucial to maintaining the immune homeostasis (146).

Furthermore, it has been observed that Tregs suppress the TCR-mediated proliferation and IL-2 release of Tconv cells (132). The mechanism by which Tregs suppress murine (169) or human (168) Tconv proliferation can be directly mediated by immunosuppressive factors or by a contact-dependent action. Tregs can also block Tconvs indirectly by controlling the activation of APCs as previously described (14).

Another mechanism to control immune activation would be effector cell-death induction by Tregs. In fact, it was observed that human tTregs express Gzm A and kill activated CD4^+^ T cells and other cells by perforin-dependent mechanism (170). Another study reported a partially Gzm B-dependent inhibition of Tconv proliferation by murine Tregs *in vitro*, although perforin was not involved (130). A further example of suppression of T effector cells by Tregs was demonstrated *in vitro* and *in vivo* in a model of transplantation in mice with the involvement of the death receptor TRAIL (171).

At the same time, high-level IL-2R expression on Tregs, indispensable in Treg cell homeostasis (172), could deprive Teff of IL-2 and inhibit their proliferation (173). Nevertheless, there is a controversy about the role, if any, of the decreased availability of IL-2 because of its consumption by Tregs that exhibit high expression levels of the CD25 molecule and may depend on the specific setting and stimulation conditions of the cells (14).

#### Suppression of Eosinophils and Neutrophils by Tregs

Eosinophils are secondary effector cells involved in the pathogenesis of allergy (Figure 1B). It has been reported that Tregs can inhibit their function (174), and a negative correlation has been found between the percentage of FOXP3^+^ cells in bronchoalveolar lavage fluid (BALF) from tolerant mice and the number of eosinophils detected in that fluid (65, 175). The mechanism by which Tregs inhibit eosinophils activation is mediated by IL-10 released by Tr1 cells (176, 177).

Additionally, Tregs might directly control allergic responses by induction of neutrophil apoptosis and/or by promoting an immunosuppressive phenotype on these cells (Figure 2) that generate IL-10, TGF-β1, IDO, heme oxygenase-1 (HO-1), and the suppressor of cytokine signaling 3 molecule (SOCS3) (178). These anti-inflammatory neutrophils may inhibit Th17 cell induction, which depends on the presence of both TGF-β1 and IL-6 (179). Additionally, phagocytosis of apoptotic neutrophils by macrophages and DCs results in a reduction in IL-23 release by these cells, which in turn leads to lower IL-17 secretion by CD4^+^ T cells (180). Hence, cooperation of activated Tregs with neutrophils might result in inhibition of Th17 response, providing an important control of inflammatory responses. Supporting this idea, one study (181) has shown that the reduced capacity of weanling, as compared with neonatal, mice to develop inducible bronchus-associated lymphoid tissue (iBALT) in response to LPS can be reversed by the elimination of Tregs. This was associated with a high expression of IL-17A and CXCL9 and with increased neutrophilic inflammation in the lungs.

#### Suppression of B-Cells by Tregs

Activated Tregs may directly suppress effector B-cells through the release of Gzm and perforin (182). As a consequence of this, Tregs can control IgE production and the posterior mast cell-mediated inflammation. In fact, the large amounts of IL-10 and TGF-β that are secreted by Tregs drastically inhibit IgE release, although a simultaneous increase in the production of IgG4 and IgA by B-cells has been observed. This isotype unbalance has also been reported in individuals naturally exposed to large allergen doses. Thus, beekeepers with multiple stings and patients with chronic helminthic infections have tolerance mediated by IL-10 and increased levels of antigen-specific IgG4 (183). Recently, the identification of IL-10-producing B regulatory cells with immunosuppressive function has been reported; these cells may also control the inflammatory reactions mediated by T cells (184) and may participate in the generation of peripheral CD4^+^CD25^+^ cells by inducing the development or elongating the survival of such cells (146).

#### Suppression by Tregs through Expression of Effector T Cell-Specific Transcription Factors

Several researchers have suggested that as with conventional CD4^+^ helper T cells, Tregs display various phenotypically and functionally diverse subsets and that their location in different tissues is critical for their ability to interact with and regulate the different subsets of Teff (82). Tregs may modulate a Th cell subset specifically by expressing the characteristic transcription factor as well as adhesion and chemoattractant receptors of said specific subset that would target them to the same tissues and inflammatory sites (185). These comprise chemokine receptors such as CXCR3, CCR8, and CCR6, involved in the migration of cells to locations of Th1-, Th2-, or Th17-mediated inflammatory responses, as well as other general receptors such as CCR2 and CCR5 (185, 186). In this regard, it was shown that upregulation of T-bet in a Treg subset upon IFN-γ secretion was required for the control of inflammatory responses mediated by Th1 cells (187). Similarly, the expression of IRF-4 (associated with Th2 and Th17 cells) in Tregs was essential for the inhibition of immune responses mediated by Th2 cells (188). Moreover, the presence of the transcription factor STAT3, characteristic of Th17 cells, in Tregs was critical for the suppression of the intestinal inflammation associated with Th17 cells (189). In accordance with this, the absence of GATA-3, transcription factor associated with Th2 cells in Tregs, led to autoimmunity, defective FOXP3 expression, and elevated cytokine levels specific of Th1, Th2, and Th17 cells (190). In fact, Tregs lacking GATA-3 expression usually transform into a Th17 subset. In relation with that, Bcl6, which acts by suppressing GATA-3 transcriptional activity independently of IL-4 and STAT6, is crucial for the control of Th2 inflammation by Tregs (191–193). The mechanism used by Tregs to suppress each subset of Teff might include deprivation of limiting factors since Tregs accumulate at the location of the immune response. Accordingly, IRF-4 or STAT3-deficient Tregs lack suppressive function *in vitro*. In contrast, the presence of certain transcription factors in Tregs might inhibit FOXP3 expression with the subsequent blocking of Tregs suppressive function. Thus, STAT3 is a transcription factor induced by the release of various cytokines that downregulate Tregs and FOXP3, such as IL-6, IL-23, or IL-27 (192, 194). In fact, the lack of STAT3 in T cells in a model of induced colitis promoted the development of Treg and diminished the symptoms of the disease (195). Probably, the different CD4^+^ T cell subsets have the ability to experiment high levels of plasticity between them, although there is controversy about the stability of the Tregs *in vivo* (22, 196). Nevertheless, several studies indicate an evident plasticity between Th17 cells and pTregs (197), and supporting this idea, a transitory stage with simultaneous expression of RORγt and FOXP3 has been detected (198). In the context of a specific cytokine environment, the release of IL-17 in murine and human Tregs, which might maintain immunosuppressive activity, was described (199, 200).

#### Suppression by Tregs *via* Ectoenzymes

During the immune response, extracellular ATP acts as a danger signal and may exert its effects on DCs. Cell damage induces the release of the intracellular ATP since the nucleotide is present within the cells in high concentration. “*Extracellular ATP can be sensed by purinergic P2 receptors such as CD39. This molecule is the main ectoenzyme in the immune system, hydrolyzes ATP or ADP to AMP and is expressed by B cells, DCs, all mouse Treg cells, and about 50% of human Treg cells*” (201). Thus, another anti-inflammatory mechanism that may be used by Tregs could be catalytic inactivation of extracellular ATP by CD39 (201). Supporting this idea, CD39 knockout Tregs showed decreased suppressive capacities *in vitro* and *in vivo* (133). In fact, CD39 expression was suggested to identify a highly suppressive human Treg subset (202), and inhibition of Tconv proliferation by this subset could be partially abolished by suppression of ectonucleotidase activity (203). CD73 is another ectoenzyme, also expressed by Tregs, that degrades AMP to adenosine (204). Adenosine binds to the A2A receptor and may suppress DCs and/or Teff, e.g., by increasing cAMP (205). *In vivo*, signaling through A2A receptor might lead to anergy and induce pTreg development (206). In conclusion, adenosine seems to contribute to the regulatory function of certain Treg subsets. Thereby, cAMP is transferred through gap junctions from the Tregs into Teff where it activates protein kinase A that suppresses proliferation and IL-2 release by triggering the activation of inducible cAMP early repressor (ICER) (207).

#### Suppression by Tregs *via* Cytokines Secretion: TGF-β, IL-10, and IL-35

Despite the critical role of TGF-β and IL-10 in various *in vivo* models, the specific contribution of immunosuppressive cytokines in Treg-mediated regulation is still poorly understood (Figure 2).

##### Role of TGF-β

TGF-β is a pleiotropic cytokine that directly prevents proliferation of T- and B-cells and also induces cell death of immature or naive B-cells. In addition, this cytokine inhibits macrophage proliferation and function, acts such as a chemoattractant for eosinophils and can suppress allergen-specific IgE release. Furthermore, TGF-β participates in Treg function and supports the generation of pTregs. TGF-β may promote pro-inflammatory Th17 responses. Thus, TGF-β favors the conversion of Teff into FOXP3^+^ Tregs in the periphery (208). However, in the presence of IL-6, TGF-β sustains the differentiation of Th17 from Tconv (177). *In vivo* studies have demonstrated the existence of a TGF-β-dependent mechanism for Treg-mediated immunosuppression (164). In this respect, it has been shown that TGF-β1 specifically expressed by Tregs plays a role in the regulation of allergic responses. In fact, TGF-β is involved in a negative feedback mechanism to regulate airway inflammatory responses, repair asthmatic tissues, and induce fibrosis in human subjects (177). Nevertheless, the effects of TGF-β in patients with allergic disorders appear to be complex, with evidence of both disease inhibition and promotion. Tregs can release large quantities of soluble or membrane-bound TGF-β and the partial neutralization of TGF-β reversed the *in vitro* inhibition of murine and human T-cell proliferation (128, 209, 210), supporting the hypothesis that TGF-β secreted by Tregs regulates inflammatory responses. However, other researchers found no such connection between TGF-β release and suppression of T cells by Tregs (211, 212). Furthermore, neutralizing antibodies against TGF-β did not block suppressive activity *in vitro* and *in vivo*, and effector T cell responses were not inhibited by supernatants from cellular suppression assays (213). Furthermore, Tregs from TGF-β-deficient mice maintain their suppressive function (214). Probably, the contribution of TGF-β to the immunosuppressive function of Tregs might depend on the location of the immune response and the characteristics of the effector cells involved in the process.

##### Role of IL-10

IL-10 is a cytokine synthesized by a diverse number of cell types, including B-cells, monocytes, DCs, natural killer cells, and T cells. The requirement for the release of IL-10 by Tregs in the control of allergic reactions has been demonstrated. In fact, inhibition of allergic airway inflammation has been shown by adoptively transferred allergen-specific Tregs (118, 215). Supporting the concept that IL-10 produced by Tregs plays a fundamental and non-redundant role in the induction of immune tolerance in patients with allergic airway disorders, studies by several researchers show that Treg cell-specific deletion of IL-10 promoted allergic airway inflammation (195). IL-10 has immunosuppressive functions and can modulate the activity of several cell subsets involved in allergic reactions, such as mast cells (216), Th2 T cells (217), eosinophils, and DCs (149). Specifically, it decreases pro-inflammatory cytokine release and Th1 and Th2 cell response, probably due to their effects on APC. Direct effects on T-cell function have also been demonstrated. T-cell activation requires antigen-specific recognition by the TCR and the signaling through costimulatory molecules such as CD28 and ICOS. On these cells, the tyrosine kinase Tyk-2, associated with the IL-10 receptor acts as a recruitment site for Src homology domain 2-containing protein tyrosine phosphatase 1 (SHP-1), which is a negative regulator for T-cell activation. After IL-10 binds to its receptor, Tyk-2 phosphorylates SHP-1 (117, 149), which immediately binds to and dephosphorylates the CD28 and ICOS costimulatory receptors, producing the inhibition of downstream signaling (218). Accordingly, T cells from SHP-1^−/−^ mice exhibited increased activation upon CD28 and ICOS binding compared with control mice, which was not attenuated by IL-10 supply. Activation of mast cells and eosinophils, the two effector cell types involved in the early and late phases of the allergic response, were inhibited by IL-10. Data from studies in murine models and human subjects have indicated that IL-10 contributes to the immune homeostasis in lung tissue (28).

##### Role of IL-35

IL-35 is a cytokine composed of two different subunits: the Epstein–Barr virus-induced gene 3 (EBI3) and a subunit of IL-12 (p35, IL-12α). It was identified as an anti-inflammatory and immunosuppressive cytokine produced mainly by Tregs (219). According to that, Tregs lacking one of the two subunits of IL-35 had decreased suppressive capability *in vitro* and *in vivo* in an intestinal bowel disease (IBD) murine model. Furthermore, EBI3^−^/^−^ and IL-12α^−^/^−^ mice have Tregs with attenuated suppressive capacity, which supports the role of IL-35 in Treg-mediated immunosuppression. In contrast to mice, human Tregs do not constitutively express IL-35 (220), although this cytokine may contribute to human regulation. In fact, the treatment of Tconv (human or murine) with IL-35 promoted Tregs-mediated suppression and did not require IL-10, TGF-β, or FOXP3 (221). A specific role of IL-35 in allergic responses has been described. Thus, IL-35 protein and mRNA levels in allergic asthmatics were shown to be lower than in healthy controls (222). In addition, the number of FOXP3^+^ Tregs and IL-12p35^+^ T cells in patients with AA was also found to be decreased (222). Furthermore, the production of IL-17, allergic airway hyperresponsiveness and the frequencies of macrophages, neutrophils, lymphocytes, and eosinophils in BALF increased in mice that were deficient in EBI-3 (223). Finally, it has been demonstrated that IL-4, IL-5, and IL-13 in BALF were inhibited by the administration of plasmid DNA encoding recombinant single-chain IL-35 or adenovirus expressing IL-35 (224) These findings strongly support a fundamental role for IL-35 in the control of allergic responses.

#### Suppression by Tregs *via* Transfer of miRNAs

microRNAs have recently been described as critical regulators of Treg development and function. These are small double-stranded RNAs that negatively regulate gene expression at a post-transcriptional stage (225). Furthermore, an exosomal pathway has been described that can capture miRNAs from certain cells and transfer them to other cells (226), providing a mechanism for cell communication. In this sense, Okoye et al. (53) have observed that Tregs are capable of releasing miRNA-containing exosomes. Specifically, Tregs released and transferred Let7d to Th1 cells, regulating Th1 cell proliferation and IFN-γ release. Furthermore, generation of miRNA and the release of exosomes by Tregs were both required for suppression of Th1 cell activation *in vivo* and for the prevention of systemic inflammatory disorders. Supporting this new mechanism of control by Tregs, it has been demonstrated that exosomes isolated from Tregs can inhibit Teff. However, this suppressive effect was not as potent as that of Tregs, suggesting that exosome transfer and other mechanisms are necessary for optimal regulation (53). Additionally, miR-21 expression has been associated with Tregs deficient in Bcl6 which exhibit a defective ability to control Th2 inflammation by limiting the transcriptional activity of GATA-3 (193).

In summary, the mechanisms of Treg cell-mediated suppressive function open up the possibility that Tregs capture and deliver different miRNAs and other molecules to different cells at different times depending on the specific situation.

## Future Perspectives

Although many efforts have been made to uncover the mechanisms that control allergic responses, several aspects remain to be clarified.

First, special interest should be paid to the prevention of allergic diseases through the study of intrauterine and postnatal factors that influence the predisposition of individuals to suffer such diseases. In this sense, the mechanisms by which TLRs modulate the differentiation and immunosuppressive ability of thymic and peripheral Tregs during the different stages of their development deserve special attention.

Second, important questions have arisen regarding the controversy on the plasticity of Tregs. It remains to be further clarified whether the expression of transcription factors specific for each Th subset promotes the acquisition by Tregs of selective migratory characteristics and/or certain specialized suppressive capacities to effectively regulate the inflammatory response induced by each subset of Teff. Additionally, the mechanisms by which certain Tregs subsets target specific effector T cell populations with more efficient suppressive results than others should be explored.

Third, the contribution of each particular suppression mechanism to the maintenance of self-tolerance and immune homeostasis should be studied. It is necessary to elucidate whether a general suppressive mechanism used by any type of Treg at any tissue exists or, conversely, different mechanisms are used depending on the location, type of antigen, Treg subsets, and conditions of the immune responses.

Success in analyzing these cellular and molecular events, *in vitro* and *in vivo*, in rodents and in humans can reveal which factors are important in the proper differentiation of Tregs, in their plasticity, and which suppression mechanisms mediated by Tregs are adequate targets for an effective control of the immune responses.

## Author Contributions

MN-M produced the figures, collaborated in the writing, and performed critical reading of the manuscript. JM-G organized references, collaborated in the writing, and performed critical reading of the manuscript. EM-O wrote the paper and supervised figures and references.

## Conflict of Interest Statement

The authors declare that the research was conducted in the absence of any commercial or financial relationships that could be construed as a potential conflict of interest.
